# Investigating a key structural determinant of health, racism, and related social determinants of health in Massachusetts during the COVID-19 pandemic

**DOI:** 10.3389/fepid.2022.1018186

**Published:** 2022-10-28

**Authors:** Arvis E. Mortimer, Meagan J. Sabatino, Esther Boama-Nyarko, Maira Castañeda-Avila, Melissa Goulding, Clevanne Julce, Stephane Labossiere, Guadalupe Mabry, Asli McCullers, Eileen McNicholas, Ann Moormann, Elizabeth Schieber, Tubanji Walubita, Sarah Forrester

**Affiliations:** ^1^Department of Population and Quantitative Health Sciences, University of Massachusetts Chan Medical School, Worcester, MA, United States; ^2^YMCA Seattle | King | Snohomish, Seattle, WA, United States; ^3^Department of Epidemiology, University of Delaware, Newark, DE, United States; ^4^Department of Medicine, University of Massachusetts Chan Medical School, Worcester, MA, United States; ^5^Institute for Public Health and Medicine, Northwestern University Feinberg School of Medicine, Chicago, IL, United States

**Keywords:** SDOH, racism, structural determinants, COVID-19, Massachusetts

## Abstract

A disproportionate burden of the ongoing COVID-19 pandemic is being shouldered by members of racial and ethnic minorities and socially disadvantaged communities. Structural and social determinants of health have been recognized as key contributors to the inequalities observed. Racism, a major structural determinant of health that patterns related social determinants of health, in the USA, warrants further investigation. In this perspective piece we provide an overview of the historical context of racism, followed by preliminary findings from the ongoing COVIDStory study—a cross-sectional study addressing perceptions of COVID-19 and COVID-19 research—that highlights the experiences of non-Hispanic Black and Hispanic identifying adult participants, residing in Worcester Massachusetts, during the COVID-19 pandemic. We then discuss these findings in the context of current and past research considering racism and relevant social determinants of health. Our study results suggest that racism and its residuals (residential segregation, economic insecurity, discrimination, bias, and vigilance) are modern challenges for non-Hispanic Black and Hispanic participants, and these findings are supported by the existing literature. It is our hope that this perspective piece provides additional evidence for action on structural and social determinants affecting the health of minoritized people, especially those living in Massachusetts.

## Introduction

It is widely recognized that structural and social conditions substantially impact health outcomes (1). The ongoing COVID-19 pandemic is no exception, as many stark disparities, such as in mortality, disease severity, and hospitalization, have been linked to social determinants of health (SDOH). SDOH are the environments where people are born, live, work, play, worship, and age (2). The relationships between SDOH and the disproportionate burden of the COVID-19 pandemic on racial and ethnic minorities and socially disadvantaged groups have been explored both in Massachusetts (3–5) and around the nation (6, 7). Less emphasized, but equally as important, are the structural determinants of health that influence the distribution of SDOH (e.g., current and historical policies that shape the pattern of social determinants in populations, institutions, and cultural norms) (8).

**Figure 1:**
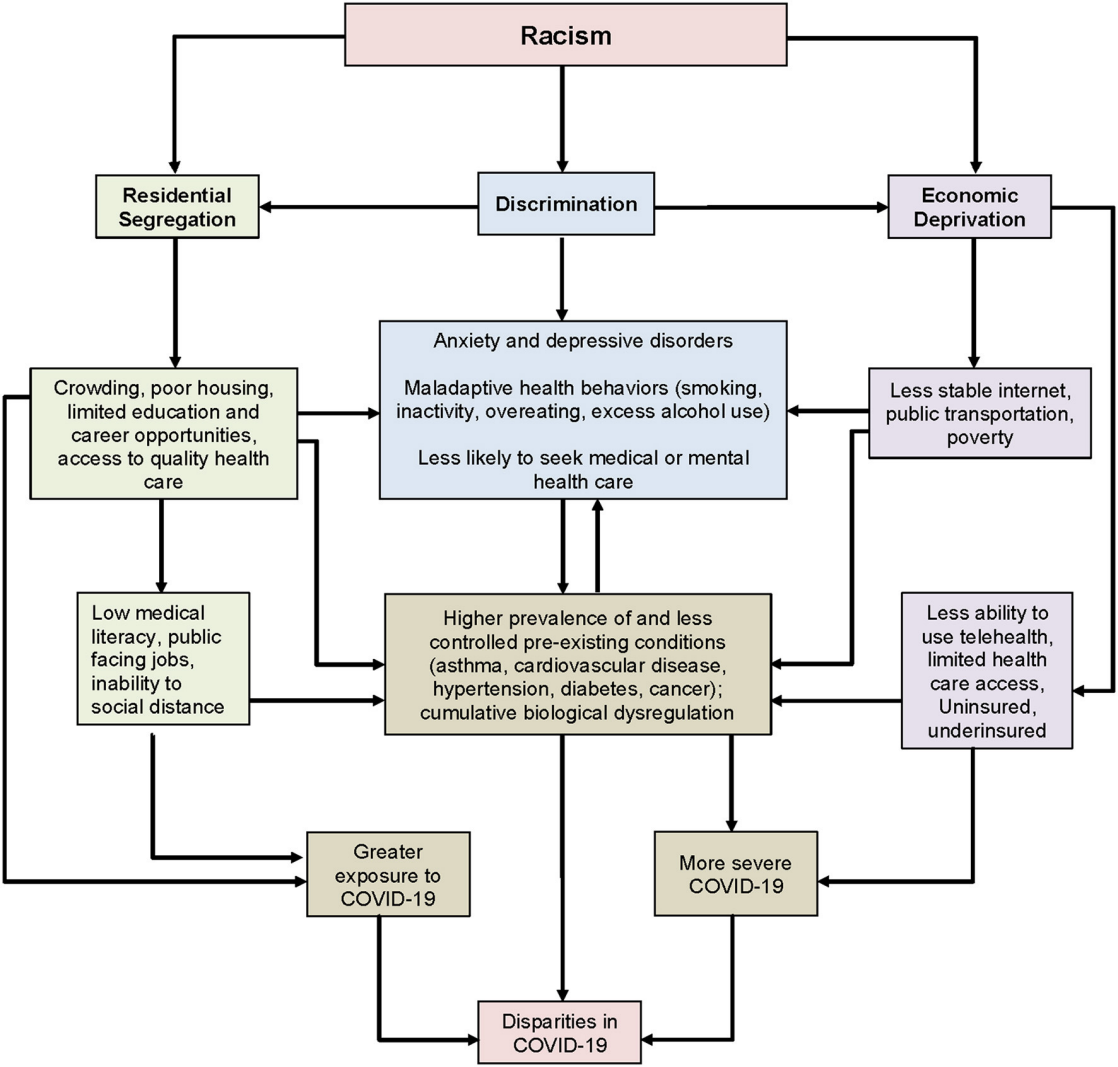
Conceptual model-Racism as root cause of disparities in COVID-19.

This paper highlights the importance of understanding and mitigating structural determinants as a means to begin equalizing SDOH in the context of the COVID-19 pandemic. We focus on the most pervasive structural determinant—racism (Figure 1). We begin by discussing the historical context of racism that shapes SDOH, we will then present preliminary descriptive data (*n* = 166) on SDOH (patterned by racism) from the COVIDStory study followed by discussions of specific SDOHs that are patterned by racism and contribute to inequality in SARS-CoV-2 transmission, morbidity, and mortality in Massachusetts. By presenting our perspective, grounded in data and an investigation of racism as a primary structural determinant, we hope to present new insights in support of action toward dismantling barriers and achieving health equity for racial and ethnic minorities.

### Historical context

One cannot fully understand and address the inequalities in American health outcomes without considering the long-lasting impacts of the United States' history. At the nation's establishment, early settlers endeavored to maintain an economy based on enslaved labor, though purporting to prioritize and uphold the rights of all “men” (9). The contradiction between the latter and legally enforced enslavement was resolved by deeming some less human. This resulted in continued systemic inequalities following emancipation (9). People of color, especially those of African descent, were placed on the lower rungs of a contrived and baseless racial ladder, which was used to justify the maintenance of widespread, oppressive systems and influenced social relations, structures, and science (9, 10).

Despite many early unjust laws and policies being formally dismantled and racial taxonomies scientifically discredited, inadequate acknowledgment, address, and remediation have led to the transformation and institutionalization of racism (9, 10). Early examples can be seen in two of Massachusetts's largest cities, Boston and Worcester. While African Americans were not legally prohibited from attending schools in the 18th and 19th centuries, unofficial bans existed. By 1830, African American abolitionists began working with White abolitionists to end segregation in schools, transportation, and other public spaces (11). Though Black persons moved to Worcester during a time of immense industrial growth—they were often only allowed to work low-skilled and low-waged jobs, earning less and therefore being at greater risk for entrapment in poverty and the adverse outcomes associated with inadequate income (9, 10, 12). This relegation, based on the early racialization of people of color, continues even after the introduction of civil rights laws in the 1960s (9–13). A clear example of this includes the passage of the Fair Housing Act in 1964, homeownership, the primary means of wealth building, which continues to be highly unequal along racial lines (14). The practice of redlining, created by the Homeowner's Loan Corporation, but used by the Federal Housing Administration, determined who would get mortgages to buy homes based on the demographics of the neighborhoods in which they wanted to live. So-called “red” areas were those with Black residents, and, to a lesser extent, migrants and the working-class. Perhaps one of the most evident and long-lasting consequences of redlining, can be seen in the disinvestment in these neighborhoods, thus leading to current residential segregation, sustained economic inequalities, and unequal SDOH (15, 16).

### Social determinants of health

The COVID-19 pandemic has exacerbated existing and historical inequities across the Commonwealth of Massachusetts (17). Neighborhoods and communities, historically under-resourced and disenfranchised, have been subject to unique exposures not encountered by other groups (17). Massachusetts leadership recognizes the importance of SDOH including residential segregation and neighborhood factors, economic insecurity, discrimination, and racial bias negatively impacting the health of its communities. However, the COVID-19 pandemic has generated a distinct and accelerated intersection of health circumstances, predisposing minoritized groups to high rates of SARS-CoV-2 transmission, COVID-19 disease severity, and mortality (18, 19). Inequitable systems, driven by racism and reinforced by factors such as adverse SDOH, have disproportionately burdened communities of color throughout the pandemic and compounded effects on racialized populations' adverse physical and mental health outcomes.

## Methods

### Study population

Survey and descriptive data were collected from an ongoing study to test a storytelling intervention to increase the participation of minoritized populations in Worcester, Massachusetts in SARS-CoV-2 antibody research. The data comes from a convenience sample (*n* = 166) of Worcester County, MA residents who identify as Black and/or Hispanic. Participants had to answer “yes” to identifying as Black or Hispanic, being over 18 years of age, and living in Worcester County. Informed consent was obtained, and the UMass Chan Medical School IRB approved the study protocols. Responses were collected between September 2021 and July 2022. (20).

### Study variables and analysis

Table 1 presents study variables for our analyses. Residential segregation and neighborhood factors were measured by the zip code participants lived in the last week. Census bureau data for each zip code was obtained from data.census.gov. Economic insecurity was evaluated based on the relationship between reported income levels within an area of concentrated poverty. Finally, measures of Discrimination, Racial Bias, and Vigilance were investigated using a) the 10-item Everyday Discrimination Scale, b) 6-item Vigilance scale, and c) 9-item COVID Bias Scale. The Everyday Discrimination Scale (EDS) is employed by health disparities researchers to describe subjective experiences of daily discrimination against minoritized persons (21). The Vigilance scale is a 6-item questionnaire assessing increased attentiveness in anticipation of victimization (22). The Coronavirus Racial Bias Scale (CRBS) is a 9-item questionnaire developed as part of the Pathways to Health Study (23) assessing participants' beliefs about how the COVID-19 pandemic negatively affects societal attitudes toward people of their racial/ethnic identity. All economic and COVID discrimination-related questions were used from the PhenX Toolkit COVID-19 Protocol Library. The EDS and Vigilance Scale were used from the Measuring Discrimination Resource (22, 24).

**Table 1:** Study questions and variables for analysis.

**Residential segregation/neighborhood factors**
Do you identify as Hispanic or Latino?
What is your race?
What is your gender?
Do you consider yourself to be: heterosexual or straight, lesbian, gay, bisexual, other, prefer not to answer
**Economic Insecurity**
What was your combined annual household income (pretax); all sources in the past year?
How would you best describe your financial situation before COVID-19?
Has your household income changed significantly since COVID-19?
How worried are you that income will negatively be impacted by COVID-19?
How worried are you that your assets will be negatively impacted by COVID-19?
Since February 2020, have you either received, applied for, or tried to apply for any of the following forms of income or assistance:
• A food pantry
In the last 12 months, the food that (I/we) bought just didn't last, and (I/we) didn't have money to get more: often true, sometimes true, never true, don't know
In the last 12 months, (I/we) couldn't afford to eat balanced meals: often true, sometimes true, never true, don't know
In the last 12 months, did (you or other adults in the household) ever cut the size of your meals or skip meals because there wasn't enough money for food?
In the last 12 months, did you ever eat less than you felt you should because there wasn't enough money to buy food?
In the last 12 months, were you ever hungry but didn't eat because you couldn't afford food?
**Discrimination, Racial Bias, and Vigilance**
You are treated with less courtesy than other people: never, less than once a year, a few times a year, a few times a month, at least once a week, almost every day
You receive poorer service than other people at restaurants and stores: never, less than once a year, a few times a year, a few times a month, at least once a week, almost every day
People act as if they think you are not smart: never, less than once a year, a few times a year, a few times a month, at least once a week, almost every day
You are called names or insulted: never, less than once a year, a few times a year, a few times a month, at least once a week, almost every day
You are threatened or harassed: never, less than once a year, a few times a year, a few times a month, at least once a week, almost every day
What do you think is the main reason for these experiences? (ancestry or national origins, race, sexual orientation, gender, some other aspect of your physical appearance, education level or income level, other)
How often do you think in advance about the kind of problems you are likely to encounter? (never, hardly ever, fairly often, very often, always)
How often do you try to prepare for possible insults before leaving your home? (never, hardly ever, fairly often, very often, always)
How often do you carefully watch what you say and how you say it? (never, hardly ever, fairly often, very often, always)
How often do you carefully observe what happens around you? (never, hardly ever, fairly often, very often, always)
How often do you try to avoid certain social situations and places? (never, hardly ever, fairly often, very often, always)
I believe the country has become more dangerous for people in my race and ethnicity: (strongly agree, somewhat agree, neither agree nor disagree, somewhat disagree, strongly disagree)
I worry about people thinking I have COVID simply because of my race/ethnicity: (strongly agree, somewhat agree, neither agree nor disagree, somewhat disagree, strongly disagree)
Most social and mass media reports about COVID create bias against people of my race/ethnicity: (strongly agree, somewhat agree, neither agree nor disagree, somewhat disagree, strongly disagree)
People of my race/ethnicity are more likely to get COVID: (strongly agree, somewhat agree, neither agree nor disagree, somewhat disagree, strongly disagree)
People of my race/ethnicity will not receive COVID healthcare as good as the care others receive: (strongly agree, somewhat agree, neither agree nor disagree, somewhat disagree, strongly disagree)
Due to COVID, I have been cyberbullied because of my race/ethnicity: (strongly agree, somewhat agree, neither agree nor disagree, somewhat disagree, strongly disagree)
Since COVID I have seen a lot more cyberbullying of people of my race/ethnicity: (strongly agree, somewhat agree, neither agree nor disagree, somewhat disagree, strongly disagree)
Negative social media posts against people of my race/ethnicity have increased: (strongly agree, somewhat agree, neither agree nor disagree, somewhat disagree, strongly disagree)
People of my race/ethnicity are more likely to lose their job because of COVID: (strongly agree, somewhat agree, neither agree nor disagree, somewhat disagree, strongly disagree)

Participant sociodemographic characteristics were examined using descriptive statistics. Comparisons were made using *t*-test for continuous and chi-square test for categorical variables.

## Preliminary findings

### Residential segregation/neighborhood factors

Forty-two percent of participants identified as Black or African American, 71% identified as Hispanic/Latino (Table 2) and 65% identified as women. Two-thirds of participants indicated that they lived in Worcester City, and 52% lived in a zip code with concentrated poverty as defined by the Census Bureau (≥20% of residents living below the poverty threshold). Although the percent of Black and Hispanic residents of Massachusetts is 9.3 and 12.8%, respectively, among respondent zip codes with concentrated poverty, percentages of Black and Hispanic residents ranged from 6 to 25.2% and 18.2 to 49.5%, respectively.

**Table 2:** Race and Ethnicity of COVIDStory Participants.

	**Total** **% (*n*)**	**Hispanic/** **Latino % (*n*)**	**Not Hispanic/** **Latino** **% (*n*)**
White	19% (31)	19% (31)	0
Black or African American	42% (70)	15% (25)	27% (44)
American Indian or Alaskan Native	2% (3)	2% (3)	0
Native Hawaiian or Pacific Islander	16% (27)	16% (27)	0
Other	14% (23)	12% (20)	2% (3)
Prefer not to Answer	7% (12)	7% (12)	0

Percentages may not equal 100 due to rounding.

### Economic insecurity

The annual income of participants who lived in zip codes with concentrated poverty (Mean (M) = $38,000, Standard Deviation (SD) = $29,000) was significantly lower than for participants who did not live in zip codes with concentrated poverty (M = $60,000, SD = $40,000), *t* (134.5) = 3.7, *p* < 0.001. The mean income of the sample was about $49,000 (range = $ 0–$150,000). Sixty-nine percent of participants indicated that they had enough money to pay the bills pre-COVID and 21% indicated that they did not. However, 55% said that their household income decreased significantly since the pandemic began, 45% were very worried that the pandemic has negatively impacted their household income, and 53% were very worried that the pandemic has negatively affected the value of their assets. Fifty-five percent had low or very low food security at the time of the survey and 34% received food from a food pantry.

### Discrimination, racial bias, and vigilance

Seventy-four percent of participants indicated that they had experienced discrimination at least once a year and of those who experienced discrimination, 61% attributed the discrimination to their race or skin color. Among those who indicated that they experienced discrimination, 26% said that they exhibited vigilance behaviors (e.g., thinking in advance about problems, prepare for insults) fairly often, very often, or always. Pertaining to COVID-19 specific bias and in reference to people of the respondent's race/ethnic groups, 44% agreed that the country is more dangerous; 62% agreed that they are more likely to lose their job; and 50% agreed that they would not receive healthcare as good as the care received by other groups.

## Discussion

In this study, more than half of the participants lived in a zip code of concentrated poverty. More than 1 in 3 identified as Black or African American and more than 1 in 2 identified as Hispanic or Latino. Since the pandemic began, greater than half of participants stated that their household income significantly decreased, and more than half were reportedly food insecure. About 3 in 4 participants experienced discrimination, with a majority attributing that discrimination to their race or skin color, and a quarter very often, or more often, exhibited vigilant behaviors. This data on residential segregation and neighborhood factors, economic insecurity, and discrimination exemplifies the SDOH in a local context. While we did not investigate causality, these data supplement the growing body of evidence concerning racism and health disparities that were further magnified by the COVID-19 pandemic (15, 17, 19, 20).

### Residential segregation/neighborhood factors

The housing policies discussed in the Historical Context section continue to negatively affect the health and economic prospects of those shunted or confined to neighborhoods deemed undesirable. In this study, we observed that more than half of participants live in a zip code of concentrated poverty. Evidence shows that even before the pandemic, historical housing policies were associated with the health of residents in segregated and disinvested neighborhoods (25, 26). Physical separation of races into neighborhoods, or residential segregation, has repeatedly been shown to negatively affect the health of residents through concentrated poverty, limited access to quality care, and limited access to health-maintaining behaviors (e.g., fresh foods, physical activity) (27). These pre-existing barriers have resulted in a disproportionate impact of the COVID-19 pandemic on those who live in disadvantaged neighborhoods. Since the beginning of the pandemic, studies have illustrated this in major cities such as New York and Washington, DC (15, 28). Similar trends in multiple states throughout the USA have been observed (29). Recent literature highlights that increased accessibility to a testing location resulted in a reduced risk for COVID-19 infection (30). In Massachusetts residential segregation of Hispanic and Black Americans was associated with a higher COVID-19 incidence rate, even though these same populations had the shortest drive time to testing sites (30). Results highlighted that SDOH such as poverty, road density, and closeness at home (more than one person to a room) were explanatory factors for these associations. As previously mentioned, one of the primary ways of wealth building in the US is through home equity meaning that those who were unable to buy homes had less opportunity to build generational wealth. This leads to less economic security in populations that were left out along racial lines. Segregated neighborhoods tend to have a higher density of people, a key driver of SARS-CoV-2 transmission. Although research has shown that living in segregated neighborhoods is associated with poor health outcomes, individual economic circumstances also play a role. Such neighborhood conditions further racial inequalities and have contributed to COVID-19 related disparities.

### Economic insecurity

There is a strong bidirectional association between economics and health outcomes (31). From our preliminary findings, a significant proportion of participants had substantial decreases in their income, were concerned with job loss, and experienced food insecurity due to the pandemic. Economic instability increases vulnerability to adverse events by directly impacting the conditions required for a person's functioning in society (31). Adverse health outcomes make people more vulnerable to economic instability (31, 32). In the US, lack of employment endangers an individual and family's financial stability, and often results in the loss of health insurance—a pernicious combination—especially considering the significance of comorbid health conditions affecting COVID-19 outcomes (31, 32). Individuals with low incomes or economic instability are at increased risk for SARS-CoV-2 infection and the most severe disease outcomes (33–35). Furthermore, minoritized and racialized groups have experienced the greatest health and economic burden in part due to limited resources (33–35). In the US, the economic consequences of the pandemic have disproportionately harmed people of color, young adults, women, parents of young children, and low-income workers, exacerbating existing pre-pandemic disparities in accessing nutritious food, health care, and childcare (35). The disparate health outcomes related to type of employment are particularly disturbing (36, 37). Five times as many low-wage workers lost their jobs compared to middle-wage workers, while high-wage earners secured more jobs in the first year of the pandemic (35). Low-income earners also reported experiencing greater stress and barriers to mental health care compared to high-income earners (34). Low-wage workers who retained their jobs were at an increased risk for viral exposure, likely due to being declared part of the public facing workforce making social distancing less possible (33, 35). In the early months of the pandemic, it was reported that about 25% of adults in the USA had difficulty paying their bills—having to access savings or borrow money from friends and family to meet basic needs (33, 37). Increasing debt and continued unmet needs, especially among the economically disadvantaged, may further widen pre-existing disparities. Such disparities may have lasting multigenerational effects as the United States Census Bureau reported that the COVID-19 pandemic increased the number of Americans living in poverty, especially children (38). Child poverty has outsized effects not only on potential morbidity or mortality from COVID-19 but also on those children's health throughout their lifetime. Clearly, economic instability stemming from racism has long affected the health of minoritized individuals in the United States which has contributed to inequities in the burden of the COVID-19 pandemic for these populations.

### Discrimination, racial bias, and vigilance

How individuals relate to their communities is important for understanding inequities in COVID-19 outcomes. In our study, conducted during the pandemic, the majority of participants experienced discrimination and attributed that discrimination to their race or skin color, with a portion exhibiting vigilance behaviors. For centuries, discrimination and racial bias have plagued minoritized communities and it is well known that they are associated with inequities in many outcomes such as cardiovascular disease (39, 40). The pandemic has amplified discrimination and bias for already at-risk populations. Because of this, multiple scales were created and adapted to capture and understand the associations between racial/ethnic identity and physical and mental health outcomes associated with COVID-19. The Coronavirus Racial Bias Scale (CRBS) has revealed that American Indian/Alaskan Native, Asian, Black, and Hispanic/Latinx respondents believed the COVID-19 pandemic negatively affected societal attitudes toward people of their racial/ethnic identity compared to their White counterparts (23). The Everyday Discrimination Scale EDS has been adapted and framed for diverse populations in an effort to understand relationships between social factors and COVID-19 inequities (21, 24). For example, Liu et al. (34) used a version of the EDS that was adapted to ask if respondents had the discrimination experiences “due to people thinking they might have the coronavirus” (41). They found that Black and Asian participants were more likely to perceive COVID-19-related discrimination than other racial/ethnic groups and that perceiving discrimination was associated with increased mental distress. Discrimination disadvantages the physical and mental health of minoritized populations both in the near term, by eroding the trust that is important to providing quality health care and medical information, and in the longer term through vigilance and hypervigilance among those affected.

Vigilance and hypervigilance, defined as increased attentiveness and calculated behavior in anticipation of looming victimization, are often catalyzed by the effects of racial discrimination (20, 42). The COVID-19 pandemic has resulted in heightened vigilance for many individuals—specifically, groups disproportionately targeted due to racist beliefs and xenophobic ideologies (43). Coping styles related to vigilance and their impact on health are well documented. Individuals who perceive a strong need to remain vigilant in a given environment find themselves tense, worried, and often avoiding places and interactions where they suspect discrimination might be pervasive (24, 43). Qualitative findings posit that members of Black and Asian communities, in particular, identified a need to be more cautious and on “high alert” due to concerns of being threatened or attacked within the first few months of the pandemic (42). Further, research has shown widespread vigilance around becoming the target of racial discrimination in Black and Asian communities and association of such vigilance with increases in symptoms of anxiety and depression (43). Racial discrimination has multiple effects on the health of populations that experience discrimination both independently and through intervening variables such as health behaviors, utilization of health care, and mental and physical health.

## Conclusion and policy recommendations

The COVID-19 pandemic has further highlighted the macro-level factors, such as economic, physical, and social structures, which broaden the disparities gap and play a significant role in population health. Going forward, policies that focus on racial equality and reducing disparities driven by racism are needed to improve health outcomes for minoritized populations. Over the last 2 years, hundreds of policies were put into place at a local, state, and federal level to theoretically reduce the burden of the pandemic (44). Examples of these policies include the eviction moratorium, increases in SNAP benefits, the CARES Act, the reduction of federal interest rates, the continuation of public insurance eligibility coverage, the lifting of telehealth restrictions, and increasing broad-band internet access (44, 45). Armed with the disconcerting evidence of the upstream causal pathways linked to significant health disparities, policymakers now face the overwhelming task of unwinding and either terminating or adopting a form of implemented policies (44). The relentless effect of the pandemic on socioeconomically disadvantaged communities has incited an investment in health equity response efforts to avoid the expansion of long-standing disparities and triggered an interest in broad policy reform (45–47). Both within and external to the healthcare system, comprehensive actions must be taken to address the origin of these disparities, racism, and build a health equity scaffold to avoid injustices in future inevitable pandemics (44, 45, 47). Previously published research has suggested actions, including cross-sector agency collaboration by government leaders, expansion of insurance coverage, implementation of value-based payment arrangements incorporating measures of SDOH, and the incorporation of affected communities in the policy development and implementation processes, for equity-focused policy reforms (45). We hope that the momentum gained during the COVID-19 pandemic to dismantle barriers to better population health outcomes will continue.

## Data availability statement

The raw data supporting the conclusions of this article will be made available by the authors, without undue reservation.

## Ethics statement

The portions of this study involving human participants were reviewed and approved by the UMass Chan Medical School Internal Review Board (study protocol #s H00023082 and H0023083). Participants were provided electronic informed consent to enroll in this study.

## Author contributions

AM, MS, EB-N, MC-A, MG, CJ, SL, GM, AM, EM, and TW made substantial contributions to the conception and design of the work, drafted the work, provided approval for publication for the content, and agree to be accountable for all aspects of the work in ensuring that questions related to the accuracy or integrity of any part of the work are appropriately investigated and resolved. SF made substantial contributions to the conception and design of the work, revised the work critically for important intellectual content, provided approval for publication for the content, and agree to be accountable for all aspects of the work in ensuring that questions related to the accuracy or integrity of any part of the work are appropriately investigated and resolved. AM made a substantial contribution to the acquisition and interpretation of data for the work, revised the work critically for important intellectual content, provided approval for publication for the content, and agree to be accountable for all aspects of the work in ensuring that questions related to the accuracy or integrity of any part of the work are appropriately investigated and resolved. All authors contributed to the article and approved the submitted version.

## Funding

SF and AM time and the COVIDStory data are funded through the National Cancer Institute grant number 1U01CA261276-01 MS and MC-A time is funded through the National Cancer Institute grant number 5T32CA172009-08 CJ and SL time is funded through the National Institutes of Health grant number T32GM135701. Open access fees are paid from the COVIDStory Grant (1U01CA261276-01). The funders did not have any input in study design, analysis, or manuscript preparation.

## Conflict of interest

The authors declare that the research was conducted in the absence of any commercial or financial relationships that could be construed as a potential conflict of interest.

## Publisher's note

All claims expressed in this article are solely those of the authors and do not necessarily represent those of their affiliated organizations, or those of the publisher, the editors and the reviewers. Any product that may be evaluated in this article, or claim that may be made by its manufacturer, is not guaranteed or endorsed by the publisher.

## References

[1] Braveman P Egerter S Williams DR. The social determinants of health: coming of age. Annu Rev Public Health. (2011) 32:381–98. 10.1146/annurev-publhealth-031210-101218 21091195

[2] WHO Commission on Social Determinants of Health World Health Organization. Closing the Gap in a Generation: Health Equity through Action on the Social Determinants of Health: Commission on Social Determinants of Health Final Report. Geneva: World Health Organization (2008).

[3] Arcaya MC Nidam Y Binet A Gibson R Gavin V. Rising home values and Covid-19 case rates in Massachusetts. Soc Sci Med. (2020) 265:113290. 10.1016/j.socscimed.2020.113290 32843186 PMC7420948

[4] Figueroa JF Wadhera RK Lee D Yeh RW Sommers BD. Community-level factors associated with racial and ethnic disparities in Covid-19 Rates in Massachusetts: study examines community-level factors associated with racial and ethnic disparities in Covid-19 rates in Massachusetts. Health Aff. (2020) 39:1984–92. 10.1377/hlthaff.2020.01040 32853056 PMC8928571

[5] Hawkins D. Social determinants of Covid-19 in Massachusetts, United States: an ecological study. J Prevent Med Public Health. (2020) 53:220. 10.3961/jpmph.20.256 32752590 PMC7411251

[6] Abrams EM Szefler SJ. Covid-19 and the impact of social determinants of health. Lancet Respirat Med. (2020) 8:659–61. 10.1016/S2213-2600(20)30234-4 32437646 PMC7234789

[7] Dalsania AK Fastiggi MJ Kahlam A Shah R Patel K Shiau S. The relationship between social determinants of health and racial disparities in Covid-19 mortality. J Racial Ethnic Health Disparit. (2022) 9:288–95. 10.1007/s40615-020-00952-y 33403652 PMC7785288

[8] Crear-Perry J. Correa-de-Araujo R, Lewis Johnson T, McLemore MR, Neilson E, Wallace M. Social and structural determinants of health inequities in maternal health. J Women's Health. (2021) 30:230–5. 10.1089/jwh.2020.8882 33181043 PMC8020519

[9] Bailey ZD Krieger N Agénor M Graves J Linos N Bassett MT. Structural racism and health inequities in the USA: evidence and interventions. Lancet. (2017) 389:1453–63. 10.1016/S0140-6736(17)30569-X 28402827

[10] Egede LE Walker RJ. Structural racism, social risk factors, and Covid-19—a dangerous convergence for black Americans. New England J Med. (2020) 383:e77. 10.1056/NEJMp2023616 32706952 PMC7747672

[11] Kousser JM. Supremacy of equal rights: the struggle against racial discrimination in antebellum massachusetts and the foundations of the fourteenth amendment. Nw UL Rev. (1987) 82:941.

[12] O'Donovan SE. First Fruits of Freedom: The Migration of Former Slaves and Their Search for Equality in Worcester, Massachusetts. Milton Park: Taylor & Francis (2011). p. 1862–1900. 10.1080/0144039X.2011.626230

[13] Boffice A. Media Analysis of City-Wide Local News Portrayals of Youth Criminality: Worcester, Massachusetts. (2018).

[14] Choi JH McCargo A Neal M Goodman L Young C. Explaining the Black-White Homeownership Gap. Washington, DC: Urban Institute Retrieved March. (2019) 25:2021.32705568

[15] Li M Yuan F. Historical redlining and resident exposure to Covid-19: a study of New York City. Race Soc Probl. (2022) 14:85–100. 10.1007/s12552-021-09338-z 34178163 PMC8212581

[16] Mitchell B Franco J. Holc “Redlining” Maps: The Persistent Structure of Segregation and Economic Inequality. (2018).

[17] Rivera L Granberry P Estrada-Martínez L Uriarte M Siqueira E Linde-Arias AR. Covid-19 and Latinos in Massachusetts. (2020).

[18] Massachusetts Department of Public Health. Social Determinants of Health Massachusetts Commonwealth of Massachusetts. (2022). Available online at: https://www.mass.gov/social-determinants-of-health-data (accessed August 9, 2022).

[19] Sequist TD. The disproportionate impact of Covid-19 on communities of color. NEJM Catalyst Innovat Care Delivery. (2020) 1:1–9.33742351

[20] Chae DH Yip T Martz CD Chung K Richeson JA Hajat A. Vicarious racism and vigilance during the Covid-19 pandemic: mental health implications among Asian and Black Americans. Public Health Rep. (2021) 136:508–17. 10.1177/00333549211018675 34034574 PMC8203039

[21] Pascoe EA Smart Richman L. Perceived discrimination and health: a meta-analytic review. Psychol Bull. (2009) 135:531. 10.1037/a0016059 19586161 PMC2747726

[22] Clark R Benkert RA Flack JM. Large arterial elasticity varies as a function of gender and racism-related vigilance in black youth. J Adolesc Health. (2006) 39:562–9. 10.1016/j.jadohealth.2006.02.012 16982392

[23] Mays VM Cochran SD Barnes NW. Race, race-based discrimination, and health outcomes among African Americans. Annu Rev Psychol. (2007) 58:201. 10.1146/annurev.psych.57.102904.190212 16953796 PMC4181672

[24] Williams DR Yu Y Jackson JS Anderson NB. Racial differences in physical and mental health: socio-economic status, stress and discrimination. J Health Psychol. (1997) 2:335–51. 10.1177/135910539700200305 22013026

[25] Aaronson D Hartley D Mazumder B. The Effects of the 1930s Holc “Redlining” Maps. (Revised August 2020). Chicago Chicago (IL): Federal Reserve Bank (2017). 10.1257/pol.20190414

[26] Hamilton CM Strader LC Pratt JG Maiese D Hendershot T Kwok RK. The phenx toolkit: get the most from your measures. Am J Epidemiol. (2011) 174:253–60. 10.1093/aje/kwr193 21749974 PMC3141081

[27] McClure E Feinstein L Cordoba E Douglas C Emch M Robinson W. The legacy of redlining in the effect of foreclosures on detroit residents' self-rated health. Health Place. (2019) 55:9–19. 10.1016/j.healthplace.2018.10.004 30448354 PMC6345551

[28] Landrine H Corral I. Separate and unequal: residential segregation and black health disparities. Ethn Dis. (2009) 19:179. 19537230

[29] Brown KM Lewis JY Davis SK. An ecological study of the association between neighborhood racial and economic residential segregation with Covid-19 vulnerability in the United States' Capital City. Ann Epidemiol. (2021) 59:33–6. 10.1016/j.annepidem.2021.04.003 33895243 PMC9761656

[30] Hu T Yue H Wang C She B Ye X Liu R. Racial segregation, testing site access, and covid-19 incidence rate in Massachusetts, USA. Int J Environ Res Public Health. (2020) 17:9528. 10.3390/ijerph17249528 33352650 PMC7766428

[31] Marmot M. the influence of income on health: views of an epidemiologist. Health Aff. (2002) 21:31–46. 10.1377/hlthaff.21.2.31 11900185

[32] Benton A Meade E Vandenberg A. The Impact of the First Year of the Covid-19 Pandemic and Recession on Families with Low Incomes. Madison: University of Wisconsin, Institute for Research on Poverty (2021).

[33] AJN. Covid-19 job losses leave many without health insurance. AJN Am J Nursing. (2020) 120:13. 10.1097/01.NAJ.0000694504.15875.a6 32732462

[34] Liu H Chen S Liu M Nie H Lu H. Comorbid chronic diseases are strongly correlated with disease severity among Covid-19 patients: a systematic review and meta-analysis. Aging Dis. (2020) 11:668. 10.14336/AD.2020.0502 32489711 PMC7220287

[35] Winkleby M Cubbin C Ahn D. Effect of cross-level interaction between individual and neighborhood socioeconomic status on adult mortality rates. Am J Public Health. (2006) 96:2145–53. 10.2105/AJPH.2004.060970 17077398 PMC1698146

[36] Hall LR Sanchez K da Graca B Bennett MM Powers M Warren AM. Income differences and Covid-19: impact on daily life and mental health. Popul Health Manag. (2022) 25:384–91. 10.1089/pop.2021.0214 34652228

[37] Parker K Minkin R Bennett J. Economic Fallout from Covid-19 Continues to Hit Lower-Income Americans the Hardest. (2020).

[38] Center on Budget and Policy Priorities. Tracking the Covid-19 Economy's Effects on Food, Housing, and Employment Hardships. Washington, DC: (2022).

[39] OECD. Record Rise in Oecd Unemployment Rate in April 2020. News Release. Paris, France: OECD Publishing (2020).

[40] Shrider EA Kollar M Chen F Semega J. Income and Poverty in the United States: 2020. US Census Bureau, Current Population Reports (2021). p. 60–273.

[41] Fisher CB Tao X Yip T. The effects of Covid-19 victimization distress and racial bias on mental health among aian, Asian, black, and latinx young adults. Cultural Diversity Ethnic Minority Psychol. (2022). 10.1037/cdp0000539 35389692

[42] LaVeist TA Thorpe Jr RJ Pierre G Mance GA Williams DR. The relationships among vigilant coping style, race, and depression. J Social Issues. (2014) 70:241–55. 10.1111/josi.12058 24954953 PMC4061746

[43] Liu Y Finch BK Brenneke SG Thomas K Le PD. Perceived discrimination and mental distress amid the Covid-19 pandemic: evidence from the understanding America study. Am J Prev Med. (2020) 59:481–92. 10.1016/j.amepre.2020.06.007 32829968 PMC7336127

[44] Artiga S Orgera K Pham O. Disparities in Health Care: Five Key Questions and Answers. (2020).

[45] Bleser WK Shen H Crook HL Thoumi A Cholera R Pearson J. Pandemic-Driven Health Policies to Address Social Needs and Health Equity. (2022).

[46] Turner A. The Business Case for Racial Equity: A Strategy for Growth. Altarum, (2018) Contract No.: 590.

[47] White House. Executive Order on Ensuring an Equitable Pandemic Response. Washington, DC. (2021).

